# Electronic Modulation of the 3D Architectured Ni/Fe Oxyhydroxide Anchored N-Doped Carbon Aerogel with Much Improved OER Activity

**DOI:** 10.3390/gels9030190

**Published:** 2023-02-28

**Authors:** Jiaxin Lu, Wenke Hao, Xiaodong Wu, Xiaodong Shen, Sheng Cui, Wenyan Shi

**Affiliations:** 1 College of Materials Science and Engineering, Nanjing Tech University, Nanjing 210009, China; 2 Department of Electromechanical inspection, Product Quality Supervising and Inspecting Institute of Taizhou, Taizhou 225300, China

**Keywords:** carbon aerogel, Ni/Fe oxyhydroxide, nitrogen doping, OER, DFT calculations

## Abstract

It remains a big challenge to develop non-precious metal catalysts for oxygen evolution reaction (OER) in energy storage and conversion systems. Herein, a facile and cost-effective strategy is employed to in situ prepare the Ni/Fe oxyhydroxide anchored on nitrogen-doped carbon aerogel (NiFeO_x_(OH)_y_@NCA) for OER electrocatalysis. The as-prepared electrocatalyst displays a typical aerogel porous structure composed of interconnected nanoparticles with a large BET specific surface area of 231.16 m^2^·g^−1^. In addition, the resulting NiFeO_x_(OH)_y_@NCA exhibits excellent OER performance with a low overpotential of 304 mV at 10 mA·cm^−2^, a small Tafel slope of 72 mV·dec^−1^, and excellent stability after 2000 CV cycles, which is superior to the commercial RuO_2_ catalyst. The much enhanced OER performance is mainly derived from the abundant active sites, the high electrical conductivity of the Ni/Fe oxyhydroxide, and the efficient electronic transfer of the NCA structure. Density functional theory (DFT) calculations reveal that the introduction of the NCA regulates the surface electronic structure of Ni/Fe oxyhydroxide and increases the binding energy of intermediates as indicated by the d-band center theory. This work provides a new method for the construction of advanced aerogel-based materials for energy conversion and storage.

## 1. Introduction

With the development of economic globalization and the growth of the population, the exploitation of low-cost and efficient energy conversion/storage technologies has attracted widespread attention [1]. The electrolysis of water into hydrogen and oxygen has been considered one of the most effective ways to alleviate energy consumption and develop a carbon-free economy [2]. The OER (4OH^−^→2H_2_O + O_2_ + 4e^−^) process is the anodic half-reaction of the water-splitting reaction, however, it is a four-proton-electron coupling transfer process, which results in a slow kinetic process and severely impedes the energy conversion efficiency [3,4].

Noble-metal-based oxides such as RuO_2_ and IrO_2_ are usually considered commercial OER electrocatalysts. However, the applications of noble metals are limited due to their scarcity and high cost, which has restricted their large-scale production and commercialization [5,6]. Therefore, researchers are committed to developing cost-effective electrocatalysts with excellent OER performance, such as transition metal oxides [7], spinel semiconductor oxides [8], layered double hydroxides [9], metal-based oxyhydroxides [10], perovskite oxides [11], etc. Among them, transition metal-based hydroxides with low kinetic energy barriers have made great progress in structural modification and electrocatalytic properties [12,13]. In particular, Ni and Fe-containing OER catalysts are versatile substitutes for precious metal species due to their earth-abundance, low-costing, environmental benignity, and theoretically high catalytic activity. Ni/Fe oxyhydroxides are among the most active and stable electrocatalysts for OER under alkaline conditions with their performances surpassing those of Ru/Ir-based benchmark electrocatalysts, perovskite oxides spinel oxides, and other transition metal-based catalysts [14]. Ni/Fe oxyhydroxide consists of edge-shared Ni/Fe oxide octahedra layers and charge-balancing interlayer anions, and the substituted Fe^3+^ is reported to promote the OER activity of single metal (oxy)hydroxides/oxides. Fe^3+^ is generally believed to play an important role in regulating the electronic structure reconstructing the surface energy and adjusting the *OH adsorption strength on the Ni/Fe oxyhydroxide [15]. Therefore, many works related to the study of Ni/Fe-based OER electrocatalysts have been published in recent years. Kitano et al. [16] reported a Ni/Fe-based double hydroxides (ULDHs) electrocatalyst loaded with the Au cluster. The combination of gold clusters and ULDHs forms an enormous number of interfacial active sites, which initiates a dehydrogenation reaction on ULDHs to form *O intermediates and greatly increase OER performance. Wu et al. [17] employed polymeric carbon nitride (PCN) as a matrix to anchor Ni/Fe metal (NiFe@PCN), forming a Ni-Fe dual-metal sites consisting of adjacent Ni and Fe atoms coordinated with N atoms in the PCN matrix. This Ni-Fe synergistic effect leads to a much lower energy barrier and exhibits higher electrocatalytic activity than mono-metal-doped catalysts.

In addition, many studies concerning Ni-Fe catalysts deposited on carbon-based supports with a high surface area have shown superior OER activity over carrier-free Ni-Fe catalysts [18]. The electrocatalytic carrier with a high BET specific surface area can effectively prevent the agglomeration of transition metal oxyhydroxide nanoparticles and expose more effective active sites [19,20]. Moreover, its abundant pores can provide many channels for ion transport between the electrocatalyst surface and the electrolyte, thereby promoting electrolyte diffusion and increasing the reaction rate [21,22]. Faraji et al. [23] designed NiCoFe layered double hydroxide heterostructures in combination with Ti_3_C_2_ and N-doped carbon nanotubes that can exploit high electrical conductivity, abundant active sites, and strong electronic interactions to promote OER activity. Zhang [24] et al. prepared N-doped carbon-supported nickel-iron nanoparticles as an OER and HER dual functional electrocatalyst, and its outstanding catalytic activity was mainly attributed to the synergistic effect of Ni/Fe oxyhydroxide with the porous NCA, and the change of electronic structures of the adjacent carbon via N introduction [25,26].

Carbon aerogel is a typical porous material consisting of nanoparticles and polymer chains, which possesses low density, low thermal conductivity, high BET specific surface area, high porosity, and excellent self-supporting properties [27]. Therefore, it has rather promising applications in the fields of photocatalyst and electrocatalyst carriers, capacitors, and adsorbents [28]. Fu [1] et al. obtained Ni-MnO/rGO catalysts with a three-dimensional porous structure by immobilizing Ni and MnO in the network of graphene aerogels. With the multi-synergistic effects arising from Ni, MnO, and rGO aerogels, the obtained catalyst exhibited outstanding catalytic activity. Lu [29] et al. prepared Cu@Fe@Ni metal aerogels by activating the core-shell Cu@Fe@Ni through an electroactivating, which results in the migration of the Fe inner shell into the Ni shell and forming highly reactive NiOOH on the surface of the aerogel, which exhibited an enhanced OER performance.

Herein, inspired by the previous studies, we have in situ prepared the Ni/Fe oxyhydroxide on porous nitrogen-doped carbon aerogel (NiFeO_x_(OH)_y_@NCA) for OER electrochemical reaction. It exhibits excellent OER performance in alkaline media, showing an overpotential as low as 304 mV at a current density of 10 mA·cm^−2^, and a Tafel slope of 72 mV·dec^−1^, which is superior to the commercial RuO_2_. These excellent properties are mainly attributed to the coupling effect of Ni/Fe oxyhydroxide with the NCA, which can induce significant exposure to more active sites, as well as promote the charge transfer rate. To gain deep insight into the OER mechanism of the NiFeO_x_(OH)_y_@NCA, the electronic structures are studied by the DFT calculations. With the introduction of NCA into NiFeO_x_(OH)_y_, the surface electronic structures of NiFeO_x_(OH)_y_ are regulated, and the binding energy between the electrocatalyst surface and the intermediate is increased. Therefore, this work provides a simple strategy to construct low-cost and efficient electrocatalysts for applications in fuel cells, energy storage, and conversion, and other energy regeneration fields.

## 2. Results and Discussion

### 2.1. Chemical Composition and Structural Analysis

**Figure 1:**
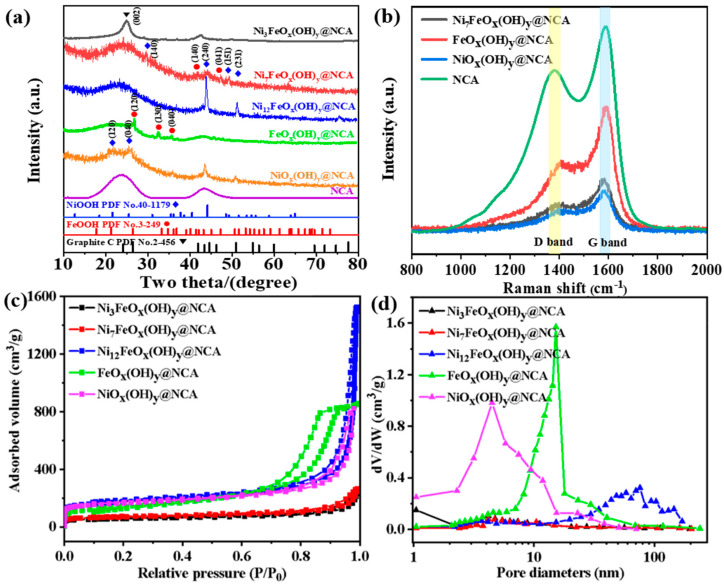
(**a**) XRD patterns, (**b**) Raman spectra, (**c**) N_2_ adsorption-desorption isotherms, (**d**) BJH pore size distribution of the resulting samples.

The X-ray diffraction (XRD) patterns of the resulting samples showing the phases and compositions are exhibited in Figure 1a. It is mentioned that FeO_x_(OH)y@NCA, and NiO_x_(OH)y@NCA are controlling samples without introducing Ni, and Fe, respectively. It is found that the broad peaks at around 26.3 ° can be ascribed to (002) crystal planes of graphite C (PDF No. 41-1487), indicating the amorphous structure of the carbon aerogel-based electrocatalyst. The peaks at 2θ = 26.5, 33.3, 36.1, 41.4 and 47.3° represent the (120), (130), (040), (140) and (041) crystal planes of FeOOH (PDF No. 3-249), respectively. In addition, it is observed that the new peaks at 2θ = 31.1, 44.1, 48.5, and 51.5° can be assigned to (140), (240), (151), and (231) planes of the NiOOH (PDF No. 40-1179) phase can be observed with the increase in Ni amount. Meanwhile, the intensities of the peaks attributed to FeOOH and NiOOH become stronger as the Ni/Fe molar ratio increases. It is worth mentioning that the intensity of the Fe and Ni species is weak, which is due to the fact that the Ni/Fe oxyhydroxide shows low crystallinity and the porous carbon support occupies most of the electrocatalyst. That is to say that the Ni_7_FeO_x_(OH)_y_@NCA sample shows an amorphous structure without strong diffraction peaks. In addition, one of the most typical characteristics of aerogel is its porous and disordered structure, therefore, we have claimed that the diffraction results indicate the typical aerogel structures. However, the existence of the Fe/Ni oxyhydroxide can be verified by TEM and XPS analysis afterward.

The Raman spectrum provides intrinsic and rich structural information about the resulting samples (Figure 1b). The resulting samples show the well-defined D-band and G-band at 1385 and 1587 cm^−1^, respectively. The D-band is associated with defects and partial disorder structures on the catalyst surface, while the G-band represents the E2g vibrational mode in the carbon domain, which shows the degree of graphitization of the resulting samples [30,31]. Generally, the peak intensity ratio of the D and G bands (I_D_/I_G_) has been considered an effective evaluation criterion for evaluating the disorder degree of carbon-based material. The estimated I_D_/I_G_ values of the three samples are extremely similar (0.582 for Ni_7_FeO_x_(OH)_y_@NCA, 0.594 for FeO_x_(OH)_y_@NCA, and 0.581 for NiO_x_(OH)_y_@NCA, respectively). This is caused by the fact that the porous NCA support occupies most of all the three electrocatalysts, with a small amount of oxyhydroxide anchored on the porous supports. Therefore, the effect of metal/metal oxide content on the degree of graphitization is extremely weak. In addition, we have also tested the I_D_/I_G_ value (0.798) of the metal-free NCA sample. It is found that as compared with the pristine metal-free NCA sample, the I_D_/I_G_ value is much decreased, showing the higher order states of NCA after the introduction of the oxyhydroxide. It is inferred that the introduction of Ni/Fe oxyhydroxide improves the graphitization of NCA, therefore increasing the charge transfer rate of the electrons during the four elementary steps, which is responsible for the much enhanced OER activity [32].

Figure 1c,d show the nitrogen adsorption/desorption isotherms and BJH size distributions of the samples with different Ni/Fe molar ratios. According to Figure 1c, the curve of FeO_x_(OH)_y_@NCA exhibits type IV isotherm with a saturated adsorption plateau, which indicates that it is typically mesoporous. The other four curves all show mixed isotherms of II and IV with H_3_-type hysteresis loops, which show no saturated adsorption plateaus, therefore, indicating the co-presence of large pores and mesopores inside the pore structures. The characteristic capillary condensation regions of the curves for the resulting samples are all in the range of 0.9–1.0, indicating that it is a typical aerogel with a three-dimensional mesoporous network structure [33,34]. It is noteworthy that the Ni_12_FeO_x_(OH)_y_@NCA sample possesses the largest adsorbed volume, and therefore it has the largest BJH adsorption pore volume of 2.20 cm^3^·g^−1^. As for the BJH distribution curves, it is noted that single-peaked pore size distributions of the FeO_x_(OH)_y_@NCA and NiO_x_(OH)_y_@NCA have been displayed. It is noted that the FeO_x_(OH)_y_@NCA has a strong peak around 15 nm, showing its rather homogeneous mesoporous structure. This indicates that the introduction of Ni has slowed down the kinetic process of the gelation and reduced the average pore size of the resulting samples. Furthermore, the most probable diameter of the Ni_12_FeO_x_(OH)_y_@NCA is around 90 nm. However, there are no distinct peaks for the Ni_3_FeO_x_(OH)_y_@NCA and Ni_7_FO_x_(OH)_y_@NCA samples. This is caused by the fact that with a large amount of Ni in the solution during the sol-gel process, the hierarchical structures including the micropores, mesopores, and large pores have formed. It is found that the optimal sample (Ni_7_FO_x_(OH)_y_@NCA) has a medial specific surface area, an adsorption pore volume, and an average pore size among the six samples (Table 1). In addition, it is worth mentioning that although the Ni_7_FeO_x_(OH)_y_@NCA sample does not have the largest BET specific surface area among the samples, it possesses the largest electrochemically active surface area (ECSA) via the electrochemical test afterward, indicating the much enhanced OER activity. The BET specific surface areas of the resulting samples are 196.07, 238.64, 614.53, 496.48, and 611.76 m^2^·g^−1^, respectively, among which the Ni_12_FeO_x_(OH)_y_@NCA and NiO_x_(OH)_y_@NCA possess extremely large BET specific surface areas larger than 600 m^2^·g^−1^. The introduction of Ni has extended the mesopores to large pore regions as indicated by Figure 1c, which can be responsible for the much-improved BET specific surface areas. It is worth mentioning that the BET specific surface area of the optimal Ni_7_FeO_x_(OH)_y_@NCA (238.64 m^2^·g^−1^) is much higher than those in the previously reported works [35,36,37], which facilitates the exposure of more active sites in the catalyst during the OER process. Meanwhile, the BJH adsorption pore volumes of the resulting samples are 0.05, 0.06, 0.14, 0.05, and 0.15 cm^3^·g^−1^, respectively. In addition, the average pore sizes are 18.05, 16.14, 32.10, 12.10, and 17.37 nm, respectively. It is noted that the optimal Ni_7_FeO_x_(OH)_y_@NCA possesses an appropriate pore size of 16.14 nm, within the middle range of the typical mesoporous aerogels, which is favorable to the enhancement of OER electrocatalytic activity.

### 2.2. Synthetic Route and Pore Morphology Analysis

**Figure 2:**
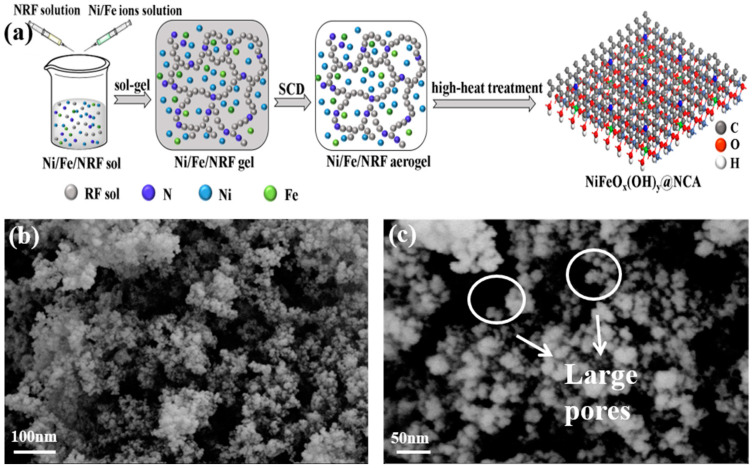
(**a**)The schematic diagram of the formation of NiFeO_x_(OH)_y_@NCA synthesized via a facile sol-gel route. (**b**,**c**) SEM images of the resulting Ni_7_FeO_x_(OH)_y_@NCA sample.

**Figure 3:**
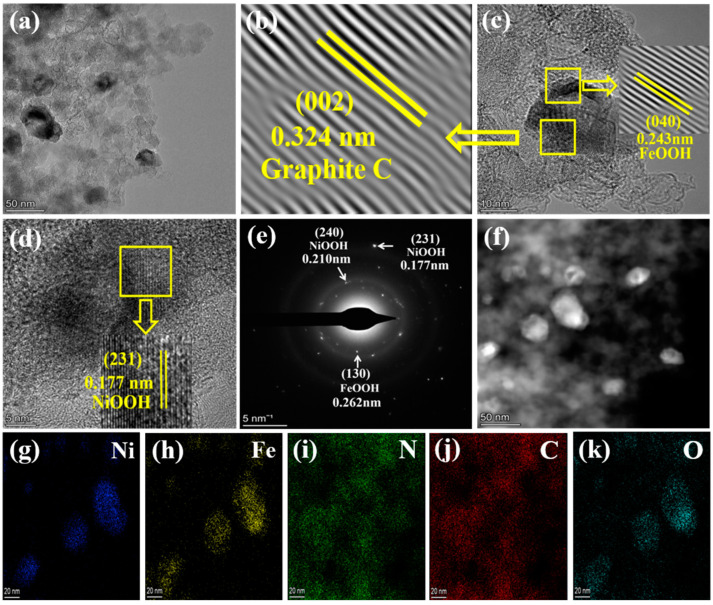
(**a**) TEM images, (**b**–**d**) HRTEM images, (**e**) SAED pattern, (**f**) STEM image with the corresponding HAADF-STEM, and (**g**–**k**) EDS mapping images of the resulting Ni_7_FeO_x_(OH)_y_@NCA.

The resulting NiFeO_x_(OH)_y_@NCA sample was obtained by in situ preparation of Ni/Fe oxyhydroxides on NCA via the sol-gel method (Figure 2a). The NiFeO_x_(OH)_y_@NCA sample was prepared by gelation, supercritical drying, and high-temperature heat treatment of the RF solution containing N-doped Ni/Fe metal salts (Ni/Fe/NRF). The Scanning Electron Microscope (SEM) images (Figure 3a,b and Figures S1 and S2) demonstrate that the resulting samples all show a 3D porous morphology composed of interconnected nanoparticles, indicating the typical aerogel structures. The FeO_x_(OH)_y_@NCA sample (Figure S1) exhibits a mesoporous and homogeneous structure with smooth morphology, and the nanoparticle diameters are at around 10–20 nm, with pore diameters of 10–30 nm. The NiO_x_(OH)_y_@NCA sample (Figure S2) has a rough surface with smaller nanoparticle diameters of 5–10 nm, and the pore sizes are located at 20–40 nm. which is consistent with the BJH pore size distribution analysis. In addition, it is noted that a small number of large pores and particle aggregation are exhibited in the as-prepared NiO_x_(OH)_y_@NCA sample due to the rich amount of Ni. As for the optimal Ni_7_FeO_x_(OH)_y_@NCA (Figure 2b,c), it is clear that the diameters of the nanoparticles are about 20–30 nm, which is between those of the FeO_x_(OH)_y_@NCA and NiO_x_(OH)_y_@NCA samples. In addition, the optimal sample shows many pores with diameters at several tens of nanometers, and it is noted that there are some large pores with diameters larger than 100 nm due to the introduction of Ni affecting grain growth. These hierarchical pore structures of the Ni_7_FeO_x_(OH)_y_@NCA aerogel provides more active sites for electron transfer, loading of the Ni/Fe oxyhydroxide, and diffusion channels for the intermediates. Therefore, the synergy effect between the N-doped carbon aerogel and Ni/Fe oxyhydroxide accelerates its OER activity.

Figure 3 presents the transmission electron microscopy (TEM) images, SAED pattern, and HADDF-STEM, as well as the EDS mapping images of the optimal Ni_7_FeO_x_(OH)_y_@NCA sample. The result (Figure 3a) shows that the sample consists of interconnected different nanocrystals of NiFeO_x_(OH)_y_ with diameters at around 20–30 nm, which is uniformly anchored on the porous carbon supports, keeping in agreement with the SEM results. The observed lattice spacing of 0.324 nm corresponds to the (002) plane of the layered graphite C, and the lattice spacing of 0.243 nm results from the (040) plane of the FeOOH phase via the high-resolution TEM (HRTEM) diagram (Figure 3b,c), respectively. In addition, the lattice spacing of 0.177 nm in Figure 3d can be attributed to the (231) crystal plane of NiOOH, which further demonstrates the formation of NiOOH and FeOOH crystalline phases in the Ni_7_FeO_x_(OH)_y_@NCA sample. The SAED pattern (Figure 3e) exhibits the (240), and (231) crystal planes of NiOOH, meanwhile, the (130) plane of FeOOH can be also observed. In addition, HADDF-STEM (Figure 3f) shows that some light spots are distributed homogeneously in the aerogel matrix, which results from the heavy Fe and Ni elements inside the resulting catalyst. Only Fe, Ni, N, C, and O elements are observed in the EDS mapping images (Figure 3g–k). It is mentioned that the distributions of Ni, Fe, and O are similar, while the distributions of N and C show the same shape. Therefore, it is further verified that the Ni/Fe oxyhydroxide has been well anchored on the surface of the NCA, which is conducive to the further improvement of its OER performance.

### 2.3. X-ray Photoelectron Spectroscopy Analysis

**Figure 4:**
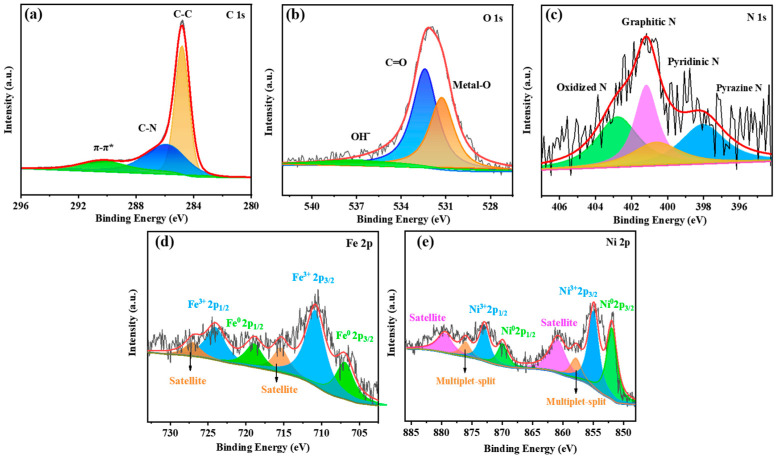
XPS spectra of (**a**) C 1s, (**b**) O 1s, (**c**) N 1s, (**d**) Fe 2p, and (**e**) Ni 2p for the optimal Ni_7_FeO_x_(OH)_y_@NCA.

XPS analysis is carried out to analyze the surface chemical composition and valence state of resulting electrocatalysts. The corresponding high-resolution C1s spectrum of the Ni_7_FeO_x_(OH)_y_@NCA sample (Figure 4a) shows the distinguishable C-C bond at 284.0 eV, the C-N bond at 285.1 eV, and the π-π* bond at 289.7 eV [38]. As shown in Figure 4b, the peaks at around 532.0 eV, 532.3 eV, and 534.0 eV can be ascribed to the metal-oxides, O-C-O, and C-OH, respectively [19,39]. As shown in Figure 4c, the peaks at around 398.1 eV, 400.6 eV, 401.2 eV, and 402.6 eV can be rationally assigned to the pyrazine N, pyridine N, graphitic N, and oxidized N, respectively [29,40]. The high-resolution XPS spectra of the Fe 2p and Ni 2p of the optimal Ni_7_FeO_x_(OH)_y_@NCA (Figure 4d,e) can be split into 2p3/2 and 2p1/2 doublets on account of the spin–orbit coupling of the electrons. The peaks at 710.8 eV and 723.9 eV can be assigned to the Fe 2p_3/2_ and Fe 2p_1/2_ of Fe^3+^. In addition, it could be observed that the peaks at 706.8 eV and 718.4 eV are caused by the Fe 2p_3/2_ and Fe 2p_1/2_ of Fe^0^ [34]. In addition, the peaks at 715.3 eV and 726.9 eV belong to the satellite peaks of Fe. As depicted in Figure 4e, the high-resolution Ni 2p spectra are consistent with previous literature reports. The peaks at 854.7 eV and 872.7 eV result from the Ni 2p_3/2_ and Ni 2p_1/2_ of Ni^3+^, and the peaks at 851.8 eV and 869.2 eV can be ascribed to the Ni 2p_3/2_ and Ni 2p_1/2_ of Ni^0^. In addition, the peaks at 857.8 eV and 876.0 eV belong to the multiplet-split of Ni^3+^2p_3/2_ and Ni^3+^2p_1/2_, and the peaks at 861.3 eV and 879.6 eV are caused by the satellite peaks of Ni [39,41], which keeps consistent with the results of the XRD, and it further confirms the successful fabrication of the NiFeO_x_(OH)_y_. The XPS spectra of C, N, and O (Figure S3a–c) for FeO_x_(OH)_y_@NCA and NiO_x_(OH)_y_@NCA samples are almost identical to the optimal Ni_7_FeO_x_(OH)_y_@NCA sample. The results obtained by the Lorentzian-Gaussian function with different contributions are concluded in Table S1. As compared with the controlling FeO_x_(OH)_y_@NCA sample, it is mentioned that the binding energy of the Fe^0^ and Fe^3+^ for the optimal sample shifts negatively, as well as the oxidized N, graphitic N, and pyrazine N peaks, which is caused by the electrons obtained from the adjacent Ni. As compared with the NiO_x_(OH)_y_@NCA sample, the binding energy of Ni^0^ and Ni^3+^ of the optimal sample both shifts negatively, which may be also caused by the introduced Fe providing rich electrons in the resulting aerogels.

### 2.4. OER Performance Analysis

**Figure 5:**
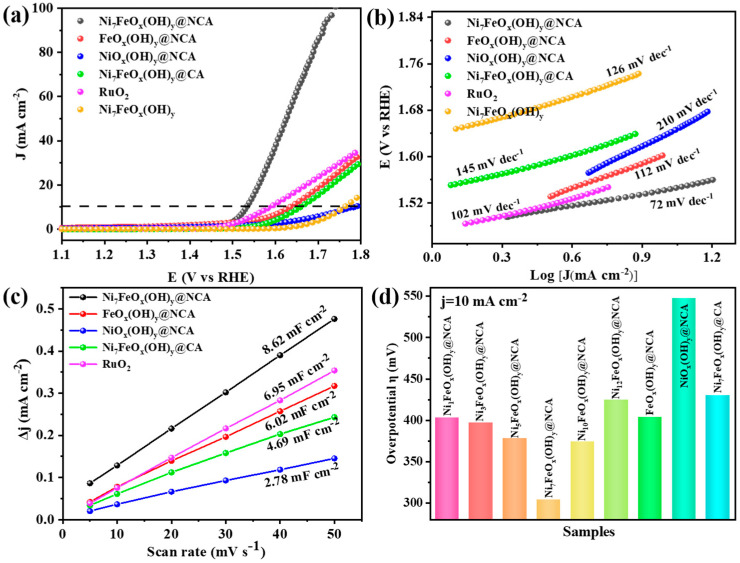
OER electrocatalytic performance. (**a**) Linear sweep voltammetry in 1.0 M KOH at a scan rate of 10 mV with iR-correction, (**b**) corresponding Tafel plots, (**c**) double-layer capacitance (C_dl_) obtained at different scan rates, and (**d**) overpotentials of electrocatalysts of the resulting samples.

The linear sweep voltammetry (LSV) curves of NiFeO_x_(OH)_y_@NCA are shown in Figure 5a. When the current density reaches 10 mA·cm^−2^, the overpotential of the Ni_7_FeO_x_(OH)_y_@NCA, FeO_x_(OH)_y_@NCA, NiO_x_(OH)_y_@NCA, Ni_7_FeO_x_(OH)_y_@CA, commercial RuO_2_, and Ni_7_FeO_x_(OH)_y_ are 304, 404, 547, 430, 356, and 532 mV, respectively. The optimal Ni_7_FeO_x_(OH)_y_@NCA shows the lowest overpotential, which is even superior to the commercial electrocatalyst RuO_2_, indicating that a proper Ni/Fe molar ratio promotes the OER property. According to the LSV curve, the performance of OER decreases sharply when there is no Fe element, therefore, we speculate that the Fe atom is likely to be the active site. Additionally, the kinetic process of the OER can be evaluated from the Tafel slope plots calculated from the polarization curves. As shown in Figure 5b, the Ni_7_FeO_x_(OH)_y_@NCA exhibits the smallest Tafel slope of 72 mV·dec^−1^, which is much better than those of the FeO_x_(OH)_y_@NCA (145 mV·dec^−1^), NiO_x_(OH)_y_@NCA (210 mV·dec^−1^), Ni_7_FeO_x_(OH)_y_@CA (112 mV·dec^−1^), RuO_2_ (102 mV·dec^−1^), and Ni_7_FeO_x_(OH)_y_ (126 mV·dec^−1^) that the optimal Ni_7_FeO_x_(OH)_y_@NCA has the fastest OER kinetics. To further investigate the electrocatalytic performance of NiFeO_x_(OH)_y_@NCA, we have performed cyclic voltammetric (CV) measurements to determine the double-layer capacitances (C_dl_), which is considered an effective evaluation method of the electronic catalytic surface area (ECSA) [42]. The CV curves of the resulting electrocatalysts at varying scan rates are shown in Figure S4. Furthermore, as shown in Figure 5c, the C_dl_ of optimal Ni_7_FeO_x_(OH)_y_@NCA is 8.62 mF·cm^−2^, which is larger than those of the FeO_x_(OH)y@NCA (6.02 mF·cm^−2^), NiFeO_x_(OH)y@NCA (4.69 mF·cm^−2^), NiO_x_(OH)_y_@NCA (2.78 mF·cm^−2^), and the commercial RuO_2_ (6.95 mF·cm^−2^). It is demonstrated that by rationally designing the structure of NiFe grown on NCA, larger active sites can be exposed. A larger ECSA favors water molecule adsorption and close contact with the electrolyte, as well as abundant active sites for electrocatalytic reactions. Figure 5d shows the overpotentials of the resulting samples, among which the optimal Ni_7_FeO_x_(OH)_y_@NCA shows excellent OER activity among the controlling samples. In addition, the effects of resorcinol (R)/Fe molar ratios on the OER electrocatalytic performance are investigated (Figure S5). It is worth noting that the R/formaldehyde (F) and the Ni/Fe molar ratio are fixed, and only various R/Fe molar ratios are carried out for the OER test. It is found that when the molar ratio of R/Fe is 80, the Ni_7_FeO_x_(OH)_y_@NCA sample shows the best OER activity among the five samples. This indicates that the OER activity is dependent on the moderate content of the Ni/Fe oxyhydroxide loaded on the NCA substrate. The effect of heat-treatment temperature on OER performance is also investigated and it is found that the electrocatalyst obtained by heat-treatment at 900 °C shows the best OER activity among the three samples (Figure S6).

**Figure 6:**
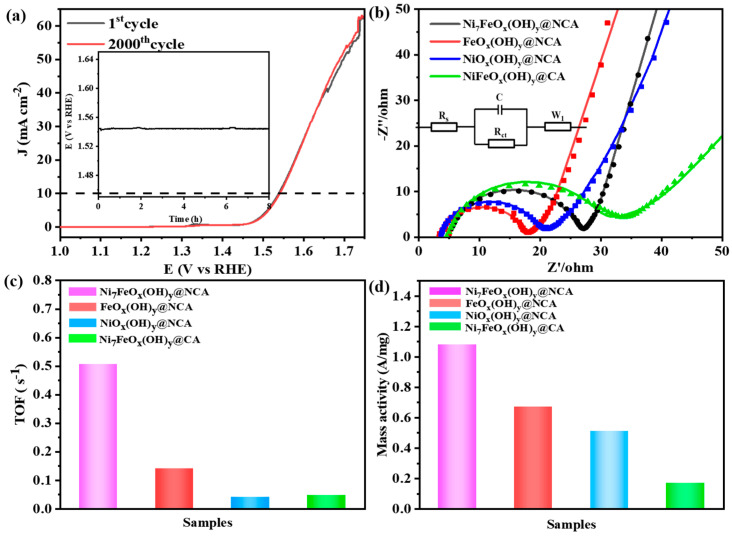
(**a**) The polarization curves recorded for the Ni_7_FeO_x_(OH)_y_@NCA before and after 2000 CV cycles (Inset: chronopotentiometric curve for the Ni_7_FeO_x_(OH)_y_@NCA at 10 mA·cm^−2^), (**b**) EIS Nyquist plots for the resulting samples, (**c**) The TOFs calculated from current at an overpotential of 300 mV, and (**d**) the mass activity of the resulting samples.

Stability is a crucial factor in assessing the feasibility of OER electrocatalytic performance. The polarization curve of the optimal Ni_7_FeO_x_(OH)_y_@NCA only slightly drifts after extended scanning of 2000 cycles (Figure 6a). The time-dependent potential profile of the Ni_7_FeO_x_(OH)_y_@NCA is also evaluated at 10 mA·cm^−2^ over 8 h (Figure 6a, inset), which further verifies that the optimal Ni_7_FeO_x_(OH)_y_@NCA is stable during the OER process. Furthermore, the SEM images of the resulting Ni_7_FeO_x_(OH)_y_@NCA aerogel catalyst after the electrochemical test is further developed to evaluate the stability. As seen in Figure S7a,b, no obvious changes including the particle size, pore size, and morphologies have been observed, further verifying the stability of the aerogel-based electrocatalyst. EIS is used to study the charge transport kinetics of the electrocatalysts. The Nyquist plots and the equivalent electrical circuit are shown in Figure 6b. The charge-transfer resistance (Rct) value of the FeO_x_(OH)y@NCA sample has the smallest Rct, which is due to its mesoporous and homogeneous structure with smooth morphology, therefore showing better electric conductivity than the optimal Ni_7_FeO_x_(OH)_y_@NCA. [43,44]. It is mentioned that the optimal Ni_7_FeO_x_(OH)_y_@NCA exhibits a proper Rct of 28.0 Ω, which is comparable with those in the previously reported literature [45,46,47], therefore, it has an excellent charge transfer property during the OER process. Furthermore, the TOF value is further tested at an overpotential of 300 mV (Figure 6c). The optimal Ni_7_FeO_x_(OH)_y_@NCA shows a large turnover frequency (TOF) value of 0.506 s^−1^, which is remarkably larger than that of FeO_x_(OH)_y_@NCA (0.167 s^−1^), NiO_x_(OH)_y_@NCA (0.067 s^−1^) and Ni_7_FeO_x_(OH)_y_@CA (0.08 s^−1^), demonstrating its rather outstanding OER activity. It is found that the Ni_7_FeO_x_(OH)_y_@NCA has the best mass activity at an overpotential of 300 mV (Figure 6d). Table S2 compares the OER activity of the optimal Ni_7_FeO_x_(OH)_y_@NCA to other similar electrocatalysts reported in the literature. Clearly, the as-prepared Ni_7_FeO_x_(OH)_y_@NCA shows strongly competitive and even better OER performance compared to the reported electrocatalysts, which can be a promising electrocatalyst for wide applications in energy saving and energy storage.

### 2.5. Theoretical Calculations

**Figure 7:**
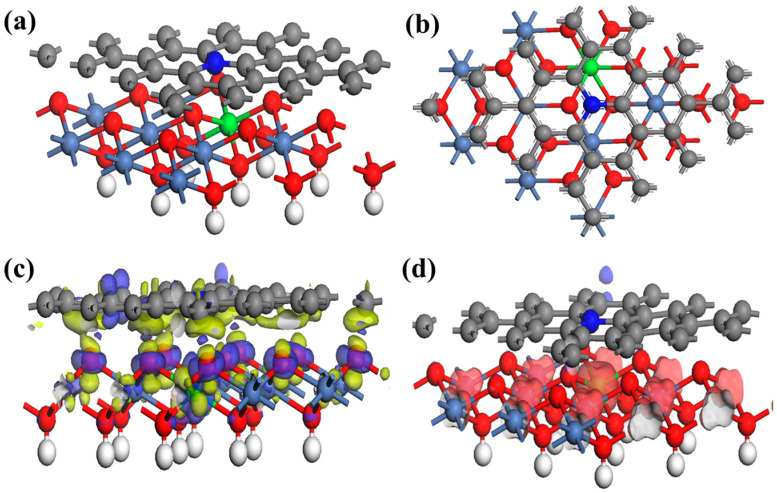
(**a**) Front view, (**b**) side view of the optimized NiFeO_x_(OH)_y_@NCA material (N: blue, C: gray, O: red, H: white, Fe: Green, and Ni: silver), (**c**) electron density difference (yellow: electron depletion and blue: electron accumulation), and (**d**) spin density field of NiFeO_x_(OH)_y_@NCA.

To gain insight into the synergistic effect of NiFeO_x_(OH)_y_ and NCA for enhancing the OER performance, the DFT calculations are further carried out. The original lattice constants of the NiFeO_x_(OH)_y_@NCA heterojunction are a = 9.28 Å, b = 9.28 Å, and c = 14.78 Å, respectively, and the lattice parameters are a = 9.93 Å, b = 9.93 Å, and c = 18.78 Å after geometry optimization. The optimized interlayer spacing is about 2.525 Å (Figure 7a,b), which verifies that a strong electronic interaction is generated between the two layers. To deeply study the electron transfer between the NiFeO_x_(OH)_y_ and the NCA layer, as well as the electron transfer within the active NiFeO_x_(OH)_y_ layer, the electron density difference (Figure 7c) is also calculated via the optimized NiFeO_x_(OH)_y_@NCA. It can be found that electron depletion occurs around the upper NCA layer and electrons accumulation occurs near the the Ni/Fe oxyhydroxide layer, indicating that electrons tend to flow from the upper NCA layer to the bottom NiFeO_x_(OH)_y_ layer, which is consistent with the XPS results. Since the spin densities of the atoms have a great impact on the OER performance of the electrocatalyst, the spin densities of the optimized structures have shown in Figure 7d. It is observed that the NiFeO_x_(OH)_y_@NCA sample echibits strong magnetism (Figure S8), mainly resulting from the center Fe atoms and the adjacent Ni also contributes a small amount of the magnetism.

**Figure 8:**
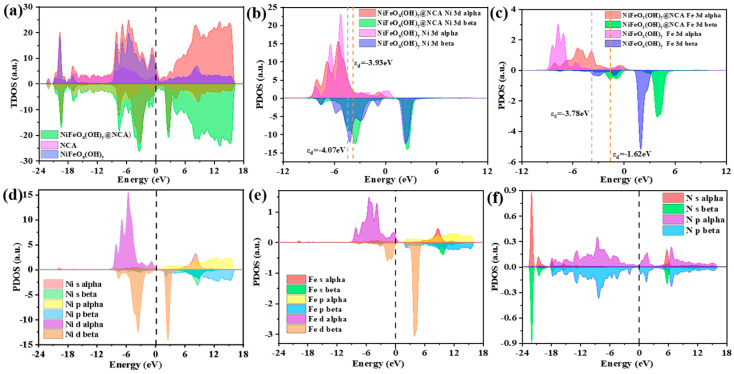
(**a**) TDOS of the individual NiFeO_x_(OH)_y_ and NCA, as well as the NiFeO_x_(OH)_y_ @NCA, d-band center calculations of the (**b**) Ni, (**c**) Fe for individual NiFeO_x_(OH)_y_ and NiFeO_x_(OH)_y_@NCA system, PDOS of (**d**) Ni, (**e**) Fe, and (**f**) N of the resulting NiFeO_x_(OH)_y_@NCA sample.

Figure 8a shows the total density of states (TDOS) and projected density of states (PDOS) of the resulting NiFeO_x_(OH)_y_@NCA, as well as the individual NiFeO_x_(OH)_y_ and NCA, which are used to study the changes in the electronic structure of the surface. Compared with individual NiFeO_x_(OH)_y_ and NCA, the NiFeO_x_(OH)_y_@NCA shows an enhanced DOS near the Fermi level due to electronic interactions, which is beneficial for charge transfer in both layers. Norskov [48] et al. have proposed that the d-band center of the metal-based active site has a significant impact on the OER performance of the electrocatalyst by tuning the binding strength of intermediates such as *OOH, *OH, and *O between the catalyst surface. According to the PDOS results shown in Figure 8b,c, the d-band center of the Ni atom of NiFeO_x_(OH)_y_@NCA positively shifts to −3.93 eV as compared to individual NiFeO_x_(OH)_y_ (−4.07 eV), and the d-band center of the Fe atom of NiFeO_x_(OH)_y_@NCA positively shifts from −3.78 eV to −1.62 eV as compared to the individual NiFeO_x_(OH)_y_. This leads to an increase in the adsorption strength of intermediates on the catalyst surface, therefore accelerating the OER performance. As shown in Figure 8d–f, it is observed that the Ni d and Fe d orbitals contribute mainly to the top of the valance band, while the bottom of the conduction band is mainly caused by the N p orbitals.

**Figure 9:**
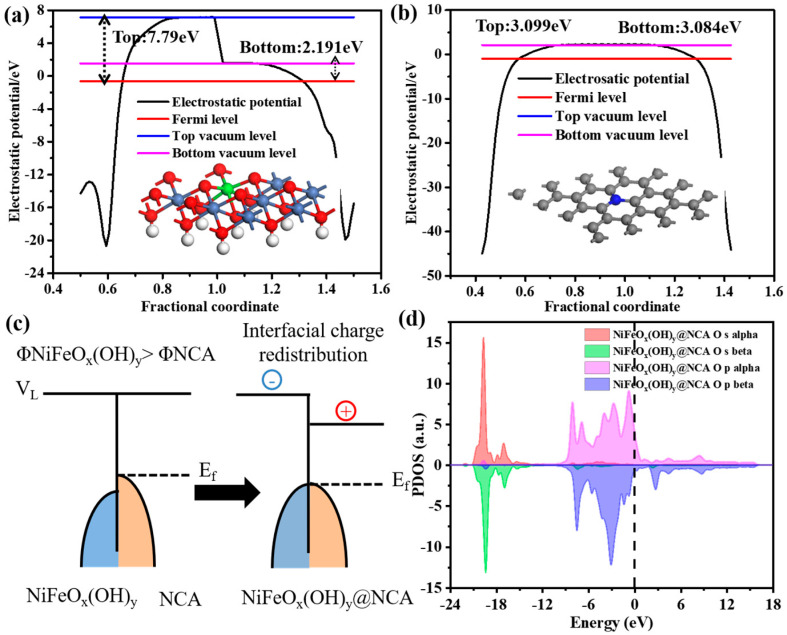
Work function of (**a**) NiFeO_x_(OH)_y_ crystal plane, and (**b**) NCA with dipole correction. (**c**) the interfacial charge redistribution of NiFeO_x_(OH)_y_ and NCA, and (**d**) the PDOS of O for NiFeO_x_(OH)_y_@NCA system.

Figure 9a,b shows the work functions of the individual NiFeO_x_(OH)_y_ (001) crystal plane and NCA. The work function of the NiFeO_x_(OH)_y_ (001) plane is 7.79 eV at the top side and 2.191 eV at the bottom side, and the NCA has a work function of 3.099 eV at the top side and 3.084 eV at the bottom side. It is worth noting that the Fermi level of NiFeO_x_(OH)_y_ is lower than NCA, which is energetic for electrons migration from NCA to NiFeO_x_(OH)_y_. Therefore, the energy bands of NiFeO_x_(OH)_y_ shift upward, while the energy bands of NCA shift downward, and finally, the two phases reach the equilibrium Fermi level (Figure 9c). Figure 9d shows the PDOS of the generated NiFeO_x_(OH)_y_@NCA sample of O. The spin-up and spin-down electronic asymmetries suggest that the strong spin densities of oxygen atoms have been induced by the adjacent Ni and Fe atoms in the resulting NiFeO_x_(OH)_y_@NCA sample, which are also responsible for the much-enhanced OER activity.

## 3. Conclusions

A novel type NiFeO_x_(OH)_y_@NCA electrocatalyst is prepared by a facile and cost-effective strategy in this work. The optimal NiFeO_x_(OH)_y_@NCA displays a typical aerogel porous structure composed of interconnected nanoparticles with a large BET specific surface area of 231.16 m^2^·g^−1^. The resulting NiFeO_x_(OH)_y_@NCA exhibits a low overpotential of 304 mV at 10 mA·cm^−2^, a small Tafel slope of 72 mV·dec^−1^, and excellent stability after 2000 CV cycles, which is superior to the commercial RuO_2_ catalyst and most of the reported OER electrocatalysts. The excellent performance is attributed to the well-designed three-dimensional structure of the carbon aerogel, the extremely large electrochemical active surface area, the synergistic effect of Ni/Fe oxyhydroxide with the porous NCA, and the change of electronic structures of the adjacent carbon via nitrogen introduction. DFT calculations show that the d-band center of the metal-based active site positively shifts and the binding strength of intermediates and the catalyst surface is greatly enhanced, which is responsible for the greatly enhanced OER activity. Therefore, this work may provide a new strategy for the development of low-cost and highly efficient carbon aerogel-based advanced electrocatalysts by tuning electronic structures.

## 4. Materials and Methods

### 4.1. Materials

Resorcinol (C_6_H_6_O_2_, 99.5%), ferric chloride hexahydrate (FeCl_3_•6H_2_O, 99.0%), and urea (CO(NH_4_)_2_) were purchased from Sinopharm Chemical Reagent Co. Ltd. Formaldehyde solution (HCHO, 37%), nickel nitrate hexahydrate (Ni(NO_3_)_2_•6H_2_O, 99.5%), and ruthenium dioxide (RuO_2_, 99.9%) were provided by Aladdin Biochemical Technology Co. Ltd. Anhydrous ethanol (EtOH, 99.7%) was provided by Wuxi Yasheng chemical reagent Co. Ltd., and deionized water (H_2_O, 99.9%) was from a Direct-Q system with a resistivity of 18.2 MΩ/cm. The above chemicals were used directly as raw materials without further purification.

### 4.2. Method

#### 4.2.1. Synthesis of NiFeO_x_(OH)_y_@NCA

The resulting NiFeO_x_(OH)_y_@NCA sample was obtained by in situ preparation of Ni/Fe oxyhydroxides on NCA via the sol-gel method. FeCl_3_·6H_2_O (27 mg), and urea (875 mg) were dissolved in the 5 mL of DI water, and then different amounts of Ni(NO_3_)_2_·6H_2_O were added into the obtained mixed solution with the Ni/Fe molar ratios of 1, 3, 5, 7,10, and 12. The above reaction mixture was further stirred for 10 min to obtain the homogeneous solution A. Resorcinol (900 mg) and formaldehyde (1.6 mL) were dissolved in H_2_O (6.8 mL) and stirred for 10 min to form a homogeneous solution B. Then, solutions A and B were mixed, and the pH value was adjusted to 7.0 with the addition of Na_2_CO_3_ (10 mg) to obtain the RF solution containing N-doped Ni/Fe metal salt (Ni/Fe/NRF sol). The solution was further stirred for 0.5 h under room temperature, and the mixture was transferred into a plastic mold until gelation was under 50 °C. The wet gels were aged at 50 °C for three days and three times each day, during which period the wet gels were washed with ethanol to remove the impurities, organic solvents, and water inside the pores. Subsequently, the wet gels were dried by CO_2_ supercritical drying to obtain the as-dried aerogels. Finally, the as-dried aerogels were carbonized under an N_2_ atmosphere at 900 °C for 2 h to obtain NiFeO_x_(OH)_y_@NCA. In addition, the FeO_x_(OH)_y_@NCA, NiO_x_(OH)_y_@NCA, and NiFeO_x_(OH)_y_@NCA samples were also produced by a similar process in the absence of Ni(NO_3_)_2_·6H_2_O, FeCl_3_·6H_2_O, and urea, respectively.

#### 4.2.2. Characterizations

The X-ray diffraction (XRD) patterns of the nanostructures were performed on a Rigaku Ultima X-ray diffractometer with Cu Kα radiation in the 2θ range of 10–80°, and the operating voltage and current were 40 kV and 40 mA, respectively, with a step size of 0.02°. X-ray photoelectron spectroscopy (XPS) measurements were performed using a Thermo Scientific K-Alpha. The XPS test was carried out with an Al Kα radiation, a spot size of 400 μm, the analyzer mode of CAE (pass energy 150.0 eV), and the step size of 1.0 eV, respectively. The microstructures and morphologies of the samples were investigated using a Scanning Electron Microscope (SEM, ZEISS Sigma 304). The N_2_ adsorption/desorption isotherms, including BET specific surface area, pore volume, and pore size distribution were performed on a V-Sorb 2800P surface area and pore distribution analyzer. Raman spectra were conducted on the Horiba Evolution equipment at an excitation laser wavelength of 532 nm. The morphology and compositions were further obtained using transmission electron microscopy (TEM, FEI TF20), and the EDS mapping images were also tested in the equipment. The magnetic properties of the resulting electrocatalyst were tested on the HH-15 vibrating sample magnetometer with a magnetic field between −10,000 to 10,000 Oe.

#### 4.2.3. Electrochemical Measurements

Electrochemical measurements were performed with an electrochemical CS2350 workstation in a three-electrode setup, using 1 M KOH as the electrolyte solution (pH = 14). The Hg/HgO (1 M NaOH) electrode and platinum wire were used as the reference electrode and counter electrode, respectively. The catalyst ink was prepared by mixing 10 mg of catalyst, 600 μL of ethanol, 400 μL of deionized water, and 40 μL 5 wt% Nafion solution, and ultrasonic for 1 h. An amount of 10 μL of catalyst ink was then loaded onto a glassy carbon electrode (3 mm in diameter), with a loading mass density of 1.37 mg/cm^2^. The linear sweep voltammetry (LSV) curves were recorded at a scan rate of 10 mV s^−1^ after electrochemical conditioning by 10 cyclic voltammetric (CV) scans reaching a stable state. The double-layer capacitances (C_dl_) for estimating the electronic catalytic surface area (ECSA) were tested by CV curves at different scan rates (10, 20, 30, 40, and 50 mV·s^−1^) in an electrochemical window of 1.175–1.215 V versus RHE. The experimental conditions for electrochemical impedance spectroscopy (EIS) was: Initial voltage (*V*) = 0, High frequency (Hz) = 100,0000, Low frequency (Hz) = 0.01, Amplitude (*V*) = 0.005, Quiet Time (s) = 2. The reference potential was calibrated to the reversible hydrogen electrode (*RHE*) based on the Nernst equation [49]:

(1) $$V_{RHE}=V_{Hg/HgO}+0.0592\times PH+0.098$$

The turnover frequency (*TOF*) was calculated from the equation [50]:

(2) $$TOF=\frac{J\times A}{4\times F\times m}$$

where *J* is the current density (*A*·cm^−2^) at a given overpotential (η = 300 mV), *A* is the surface area of the electrode, *F* is the Faraday constant (96,485 C·mol^−1^), and m is the number of moles of metal on the electrode. The mass activity (*J_m_*, A·mg^−1^) was calculated from the active mass deposited on the electrode surface (*m*, g) and the measured current *I* (*A*), as the following equation [51]:

(3) $$J_{m}=\frac{I}{m}$$

#### 4.2.4. Theoretical Calculations

Spin-polarized density functional theory (DFT) calculations were performed by the CASTEP module in the Materials Studio 8.0 package. Periodic geometry and cell optimization of NiFO_x_(OH)_y_@NCA were first performed, followed by electronic property calculations and analysis. The plane wave basis with an energy cutoff of 400 eV and ultrasoft pseudopotential was performed during all the calculations, and the exchange-correlation energy was described by the generalized gradient approximation of Perdew, Burke, and Ernzerhof (GGA-PBE) functional. To evaluate the on-site Coulomb interactions in the 3D states of NiFeO_x_(OH)_y_@@NCA hybrid, the DFT + U approach with the Hubbard parameter U = 6.45 eV, and U = 5.30 eV for Ni and Fe atom in NiFeO_x_(OH)_y_ structure. A dispersion-corrected semi-empirical TS scheme was employed to further characterize the interaction between the two layers. To simulate the structure of NiFeO_x_(OH)_y_@NCA, the (110) plane of NiOOH was selected, in which one of the eight atoms was replaced by a Fe atom, with a mismatch rate of less than 5%. The Brillouin zone was sampled through a 4×4×1 uniform k-point grid for geometric optimization and electronic structures calculations. The model structures were optimized with a total energy threshold of 10–5 eV/atom, a maximum force of 0.03 eV/Å, a maximum stress of 0.05 GPa, and a maximum displacement of 0.001 Å, respectively.

## Figures and Tables

**Table 1:** Pore structures of the resulting samples.

Samples	BET Surface Area (m^2^·g^−1^)	BJH Adsorption Pore Volumes (cm^3^·g^−1^)	BJH Adsorption Average Diameters (nm)
Ni_3_FeO_x_(OH)_y_@NCA	196.07	0.05	18.05
Ni_7_FeO_x_(OH)_y_@NCA	238.64	0.06	16.14
Ni_12_FeO_x_(OH)_y_@NCA	614.53	0.14	32.10
FeO_x_(OH)_y_@NCA	496.48	0.05	12.10
NiO_x_(OH)_y_@NCA	611.76	0.15	17.37

## Data Availability

The raw/processed data required to reproduce these findings cannot be shared at this time as the data also form part of an ongoing study.

## Supplementary Materials

The following supporting information can be downloaded at: www.mdpi.com/article/10.3390/gels9030190/s1, Figure S1. SEM images of the resulting FeO_x_(OH)_y_@NCA sample. Figure S2. SEM images of the resulting NiO_x_(OH)_y_@NCA sample. Table S1. Surface chemical compositions of the resulting samples were calculated by the Lorentzian-Gaussian function. Figure S3. XPS spectra of for (a) C 1s, (b) O 1s, (c) N 1s, (d) Fe 2p, and (e) Ni 2p for the FeO_x_(OH)_y_@NCA and NiO_x_(OH)y@NCA samples. Figure S4. CV curves of (a) Ni_7_FeO_x_(OH)_y_@NCA, (b) FeO_x_(OH)_y_@NCA, (c) NiO_x_(OH)_y_@NCA, (d) NiFeO_x_(OH)_y_@CA, and (e) RuO_2_ at potential from 0.175 V to 1.225 V vs RHE at scan rates of 5 mV/s, 10 mV/s, 20 mV/s, 30 mV/s, and 40 mV/s in 1.0 M KOH. Figure S5. LSV of the resulting Ni_7_FeO_x_(OH)_y_@NCA sample with different resorcinol/Fe molar ratios. Figure S6. LSV of the resulting Ni_7_FeO_x_(OH)_y_@NCA sample under different heat-treatment temperatures. Figure S7. The SEM images of the resulting Ni_7_FeO_x_(OH)_y_@NCA aerogel (a) before and (b) after the OER test. Table S2. Comparison of OER performance of the as-prepared Ni_7_FeO_x_(OH)_y_@NCA and other similar reported electrocatalysts. Figure S8. VSM magnetization curve of the Ni_7_FeO_x_(OH)_y_@NCA sample. References [1,3,4,5,6,7,8,9,10,11,12,13,14,15] are cited in the Supplementary Materials.
