# Supplementary material for: Electronic Modulation of the 3D Architectured Ni/Fe Oxyhydroxide Anchored N-Doped Carbon Aerogel with Much Improved OER Activity
Source: Gels. 2023 Feb 28;9(3):190. doi: 10.3390/gels9030190 (PMC10048674; doi:10.3390/gels9030190)
Supplement: Supplementary file 1 [file gels-09-00190-s001.zip › gels-2229083-supplementary.pdf]

Supplementary Information

Electronic modulation of the 3D architectured Ni/Fe oxyhydroxide anchored N-doped carbon aerogel with improved OER activity

Jiaxin Lu [1], Wenke Hao [1], Xiaodong Wu [1,*], Xiaodong Shen [1,*], Sheng Cui [1], and Wenyan Shi [2]

[1] *College of Materials Science and Engineering, Nanjing Tech University, Nanjing, 210009, China*

[2] *Product quality supervising and inspecting institute of Taizhou, Taizhou, 225300, China*

***** *Corresponding authors*

E-mail address: wuxiaodong@njtech.edu.cn;

SEM analyses of the $FeO_x(OH)_y$@NCA and $NiO_x(OH)_y$@NCA samples.

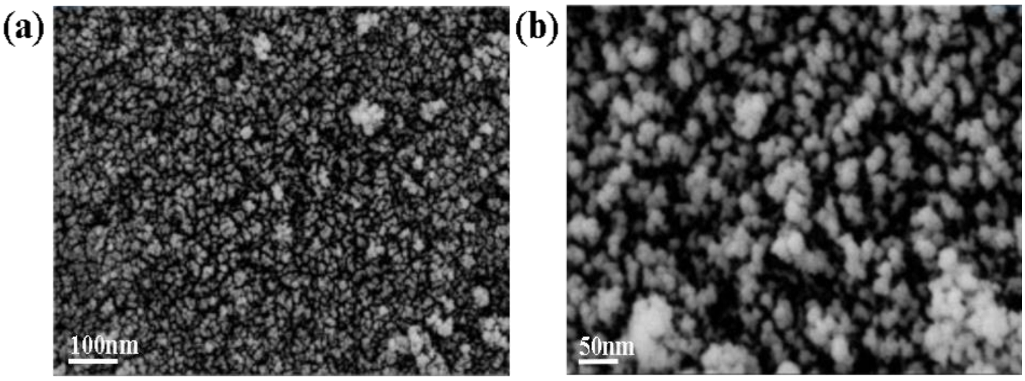

Figure S1. SEM images of the resulting $FeO_x(OH)_y$@NCA sample.

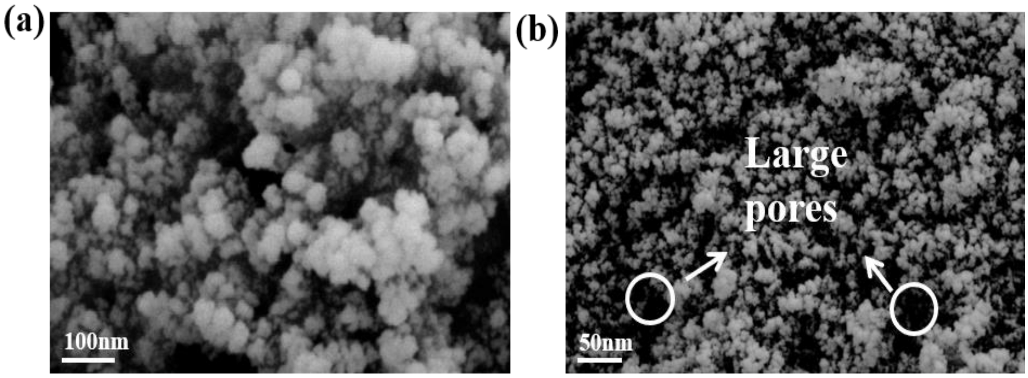

Figure S2. SEM images of the resulting $NiO_x(OH)_y$@NCA sample.

The results obtained by the Lorentzian-Gaussian function with different contributions are concluded in Table S1.

Table S1. Surface chemical compositions of the resulting samples were calculated by the Lorentzian-Gaussian function.

**Samples**	**Ni 2p /binding**	**Energy /amount**		**Fe 2p /binding**	**Energy /amount**		**N 1s /binding**	**Energy /amount**	
$Ni_7FeO_x(OH)_y$@NCA	$Ni^0\ 2p^{1/2}$	869.2	15.94 %	$Fe^0\ 2p^{1/2}$	718.4	11.95 %	Oxidized N	402.6	25.80 %
	$Ni^0\ 2p^{3/2}$	851.8	30.02 %	$Fe^0\ 2p^{3/2}$	706.8	19.27 %	Graphitic N	401.2	36.23 %
	$Ni^{3+}2p^{1/2}$	872.7	17.75 %	$Fe^{3+}\ 2p^{1/2}$	723.9	22.81 %	Pyridine N	400.6	16.41 %
	$Ni^{3+}\ 2p^{3/2}$	854.7	36.29 %	$Fe^{3+}\ 2p^{3/2}$	710.8	45.97 %	Pyrazine N	398.1	21.56 %
$FeO_x(OH)_y$ @NCA				$Fe^0\ 2p^{1/2}$	720.9	11.56 %	Oxidized N	403.3	18.09 %
				$Fe^0\ 2p^{3/2}$	707.2	20.59 %	Graphitic N	401.7	24.69 %
				$Fe^{3+}2p^{1/2}$	724.1	22.38 %	Pyridine N	401.0	34.35 %
				$Fe^{3+}\ 2p^{3/2}$	711.7	45.47 %	Pyrazine N	398.7	22.72 %
$NiO_x(OH)_y$ @NCA	$Ni^0\ 2p^{1/2}$	871.6	14.63 %				Oxidized N	403.0	20.34 %
	$Ni^0\ 2p^{3/2}$	852.9	29.45 %				Graphitic N	401.3	19.61 %
	$Ni^{3+}\ 2p^{1/2}$	873.7	17.97 %				Pyridine N	400.4	38.81 %
	$Ni^{3+}\ 2p^{3/2}$	855.3	37.95 %				Pyrazine N	398.1	21.24 %

The XPS spectra of C, N, O, Fe, and Ni for $FeO_x(OH)_y$@NCA and $NiO_x(OH)_y$@NCA samples.

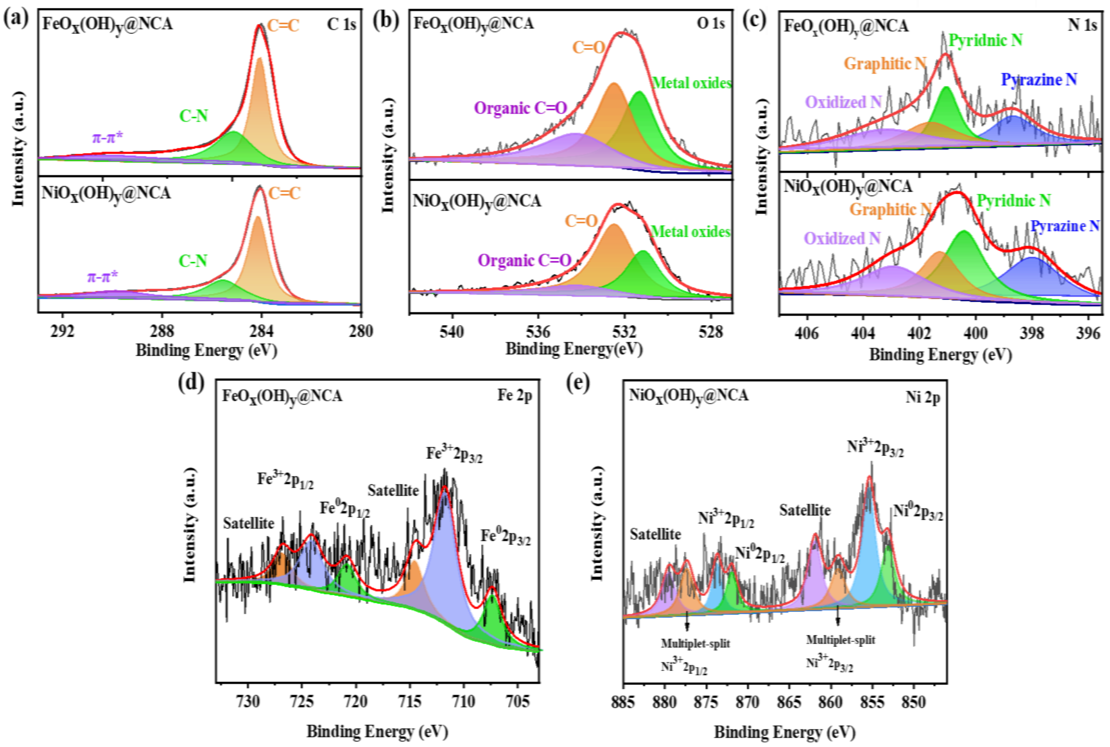

Figure S3. XPS spectra of for (a) C 1s, (b) O 1s, (c) N 1s, (d) Fe 2p, and (e) Ni 2p for the FeOx(OH)y@NCA and NiOx(OH)y@NCA samples.

The CV curves of the resulting electrocatalysts at varying scan rates.

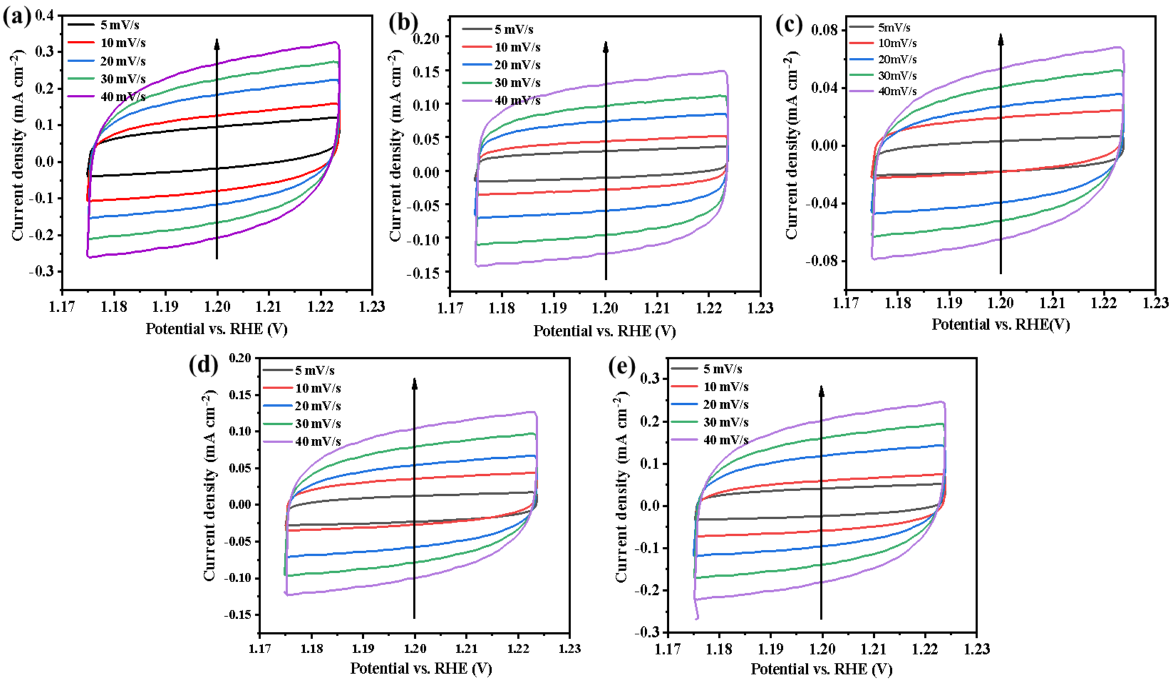

Figure S4. CV curves of (a) $Ni_7FeO_x(OH)_y$@NCA, (b) $FeO_x(OH)_y$@NCA, (c) $NiO_x(OH)_y$@NCA, (d) $Ni_7FeO_x(OH)_y$@CA, and (e) RuO_2 at potential from 0.175 V to 1.225 V vs RHE at scan rates of 5 mV/s, 10 mV/s, 20 mV/s, 30 mV/s, and 40 mV/s in 1.0 M KOH.

The LSV curves of $Ni_7FeO_x(OH)_y$@NCA with different resorcinol/Fe molar ratios.

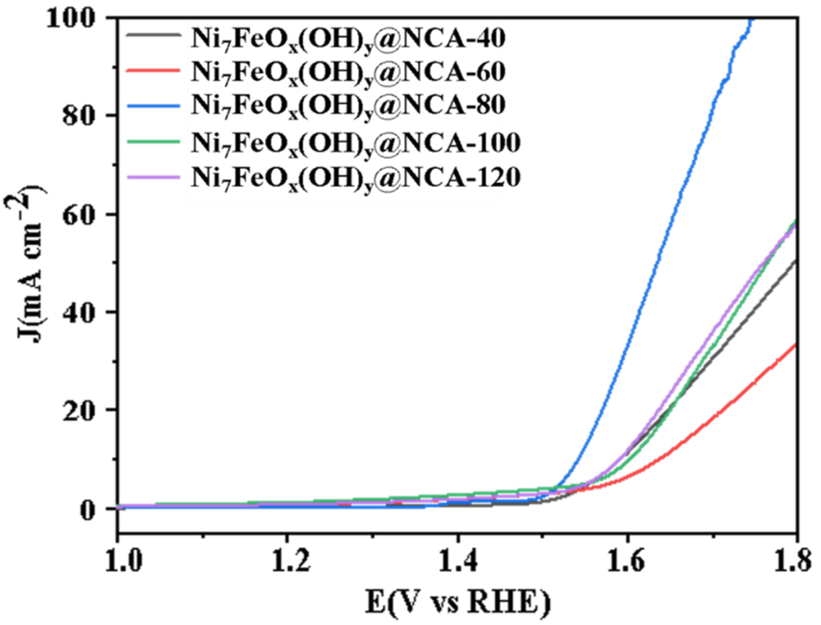

Figure S5. LSV of the resulting $Ni_7FeO_x(OH)_y$@NCA sample with different resorcinol/Fe molar ratios.

The LSV curves of $Ni_7FeO_x(OH)_y$@NCA with the different heat treatment temperatures.

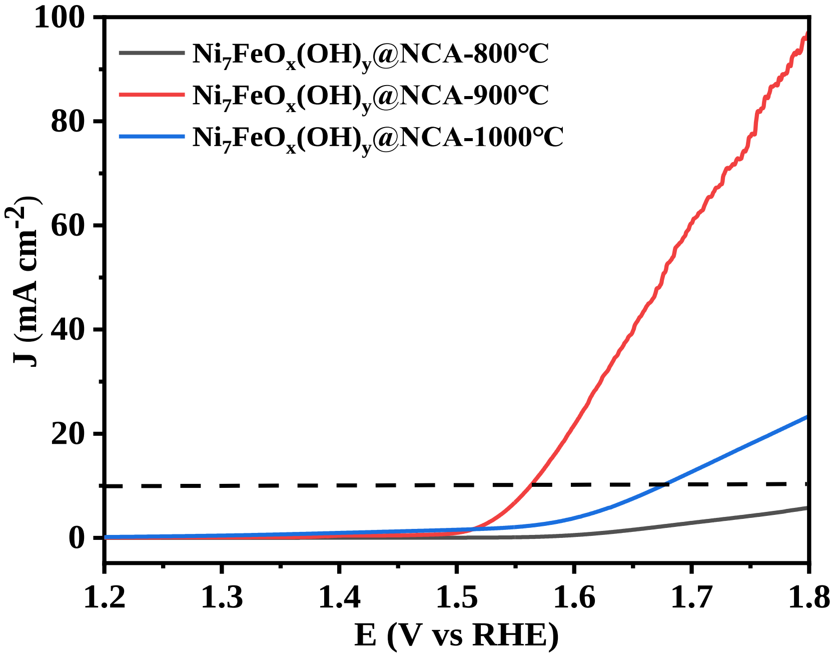

Figure S6. LSV of the resulting $Ni_7FeO_x(OH)_y$@NCA sample under different heat-treatment temperatures.

The SEM images of the resulting $Ni_7FeO_x(OH)_y$@NCA aerogel catalyst after the electrochemical test.

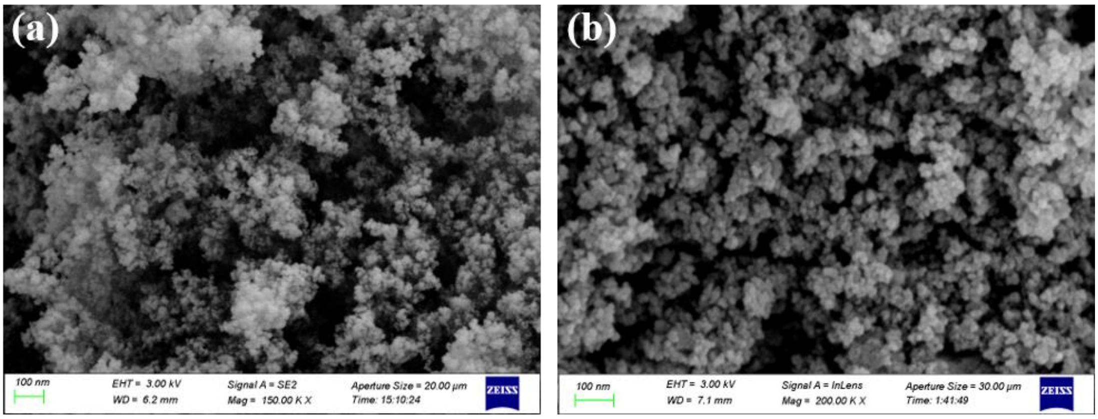

Figure S7. The SEM images of the resulting $Ni_7FeO_x(OH)_y$@NCA aerogel (a) before and (b) after the OER test.

Table S2. Compares the OER activity of the optimal $Ni_7FeO_x(OH)_y$@NCA to other similar electrocatalysts reported in the literature. Clearly, the as-prepared $Ni_7FeO_x(OH)_y$@NCA shows strongly competitive and even better OER performance compared to the reported electrocatalysts.

Table S2. Comparison of OER performance of the as-prepared $Ni_7FeO_x(OH)_y$@NCA and other similar reported electrocatalysts.

Electrocatalyst	Onset potential (mV)	η_{10} (mV)	Tafel slope (mV dec^{-1})	References
NiO/NiO(OH)	143	346	66	[1]
Ni_3FeN/r-GO	149	298	54	[2]
$Fe_{33}Ni_{33}Co_{33}$	152	322	36	[3]
TA-NiFe@NCNT	142	310	75	[4]
Ni@Ni-NC	151	371	62	[5]
NiFe–$NiFe_2O_4$	153	316	74	[6]
Ni-Fe-O_x	143	295	97	[7]
NiFe-LDH/CNT	145	299	92	[8]
NiCoP@NC/NF	147	310	90	[9]
NiFe-LDH	155	307	67	[10]
NiFe-LDH/Co,N-CNF	143	312	60	[11]
NiFe LDH/CB	145	280	35	[12]
NiFe@NC	150	350	56	[13]
NiFe alloy	153	290	53	[14]
Tannin-NiFe	153	300	28	[15]
$NiFeO_x(OH)_y$@NCA	151	304	72	**This work**

Magnetic properties of $NiFeO_x(OH)_y$@NCA samples

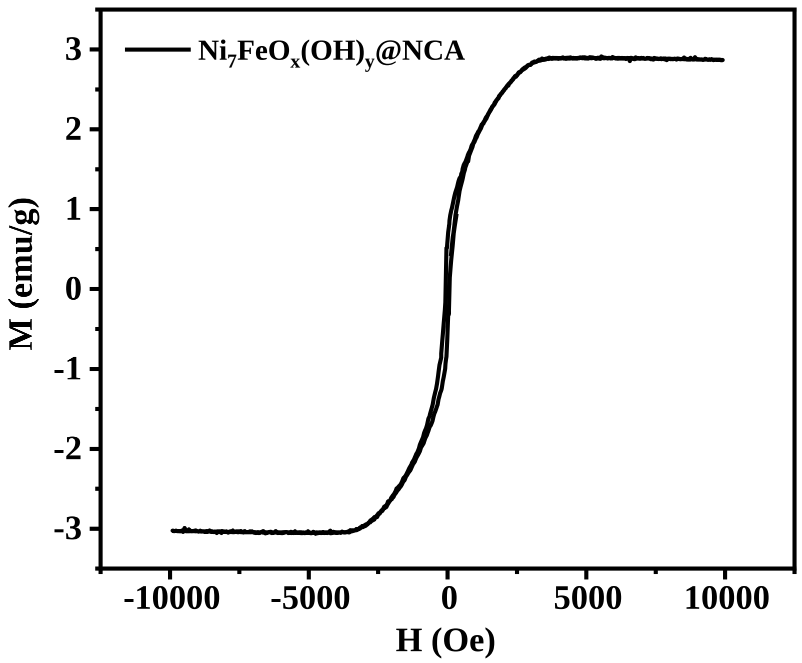

Figure S8. VSM magnetization curve of the $Ni_7FeO_x(OH)_y$@NCA sample.

References

1. Hashemi, N.; Nandy, S.; Chae, K.H.; Najafpour, M.M. Anodization of a NiFe Foam: An Efficient and Stable Electrode for Oxygen-Evolution Reaction. *ACS Appl. Energy Mater.* **2022,** 5, 11098-11112.
2. Gu, Y.; Chen, S.; Ren, J.; Jia, Y.A.; Chen, C.M.; Komarneni, S.; Yang, D.J.; Yao, X.D. Electronic structure tuning in Ni_3FeN/r-GO aerogel toward bifunctional electrocatalyst for overall water splitting. *ACS Nano* **2018,** 12, 245-253.
3. Florian, F.L.; Yerly, L.; Mensi, E.P.; Costa, X.P.D.; Boudoire, F.; Guijarro, N.; Spodaryk, M.; Züttel, A.; Sivula, K. Influence of Composition on Performance in Metallic Iron–Nickel–Cobalt Ternary Anodes for Alkaline Water Electrolysis. *ACS Catal.* **2020,** 10, 12139-12147.
4. Xie, X.Y.; Shang, L.; Shi, R.; Waterhouse, G.I.; Zhao, J.Q.; Zhang, T.R. Tubular assemblies of N-doped carbon nanotubes loaded with NiFe alloy nanoparticles as efficient bifunctional catalysts for rechargeable zinc-air batteries. *Nanoscale* **2020,** 12, 13129-13136.
5. Seok, S.; Choi, M.; Lee, Y.; Jang, D.; Shin, Y.; Kim, Y.H.; Jo, C.; Park, S.J. Ni Nanoparticles on Ni Core/N-Doped Carbon Shell Heterostructures for Electrocatalytic Oxygen Evolution. *ACS Appl. Nano Mater.* **2021,** 4, 9418-9429.
6. Raimundo, R.A.; Silva, V.D.; Medeiros, E.S.; Macedo, D.A.; Simões, T.A.; Gomes, U.U.; Morales, M.A.; Gomes, R.M. Multifunctional solution blow spun NiFe-$NiFe_2O_4$ composite nanofibers: structure, magnetic properties and OER activity. *J. Phys. Chem. Solids.* **2020,** 139, 109325.
7. Vincent, I.; Lee, E.C.; Kim, H.M. Highly Active Ni–Fe Based Oxide Oxygen Evolution Reaction Electrocatalysts for Alkaline Anion Exchange Membrane Electrolyser. *Catalysts* **2022,** 12, 476.
8. Xu, H.J.; Wang, B.K.; Shan, C.F.; Xi, P.X.; Liu, W.S.; Tang, Y. Ce-Doped NiFe-Layered double hydroxide ultrathin nanosheets/nanocarbon hierarchical nanocomposite as an efficient oxygen evolution catalyst. *ACS. Appl. Mater. Inter.* **2018,** 10, 6336-6345.
9. Nie, J.H.; Hong, M.; Zhang, X.H.; Huang, J.L.; Meng, Q.; Du, C.C.; Chen, J.H. 3D amorphous NiFe LDH nanosheets electrodeposited on in situ grown NiCoP@NC on nickel foam for remarkably enhanced OER electrocatalytic performance. *Dalton T.* **2020,** 49, 4896-4903.
10. Yu, L.; Zhou, H.Q.; Sun, J.Y.; Qin, F.; Yu, F.; Bao, J.M.; Yu, Y.; Chen, S.; Ren, Z.F. Cu nanowires shelled with NiFe layered double hydroxide nanosheets as bifunctional electrocatalysts for overall water splitting. *Energy Environ. Sci.* **2017,** 10, 1820-1827.

11. Wang, Q.; Shang, L.; Shi, R.; Zhang, X.; Zhao, Waterhouse, G.I.; Wu, L.Z.; Tung, C.H.; Zhang, T.R. NiFe layered double hydroxide nanoparticles on Co, N-Codoped carbon nanoframes as efficient bifunctional catalysts for rechargeable zinc-air batteries. *Adv. Energy Mater.* **2017,** 7, 1700467.
12. Munonde, T.S.; Zheng, H.T.; Nomngongo, P.N. Ultrasonic exfoliation of NiFe LDH/CB nanosheets for enhanced oxygen evolution catalysis. *Ultrason. Sonochem.* **2019,** 59, 104716.
13. Lai, C.L.; Gong, M.X.; Zhou, Y.C.; Fang, J.Y.; Huang, L.; Deng, Z.P.; Liu, X.P.; Zhao, T.H.; Lin, R.Q.; Wang, K.L.; Jiang, K.; Xin, H.L.; Wang, D.L. Sulphur modulated Ni_3FeN supported on N/S co-doped graphene boosts rechargeable/flexible Zn-air battery performance. *Appl. Catal. B-Environ.* **2020,** 274, 119086.
14. Yamada, N.; Kitano, S.; Yato, Y.; Kowalski, D.; Aoki, Y.; Habazaki, H. In Situ Activation of Anodized Ni–Fe Alloys for the Oxygen Evolution Reaction in Alkaline Media. *ACS Appl. Energy Mater.* **2021,** 3, 12316-12326.
15. Shi, Y.M.; Yu, Y.; Liang, Y.; Du, Y.H.; Zhang, B. In situ electrochemical conversion of an ultrathin tannin nickel iron complex film as an efficient oxygen evolution reaction electrocatalyst. *Angew. Chem. Int. Edit.* **2019,** 58, 3769-3773.
